# Anti-Neuroinflammatory Effects of Cardenolides in IL-1β-Activated SK-N-SH Cells

**DOI:** 10.3390/molecules31193501

**Published:** 2026-10-01

**Authors:** Lu Sun, Matthias Apweiler, Rami Schulzki, Christoph W. Grathwol, Claus Normann, Stefan Bräse, Bernd L. Fiebich

**Affiliations:** 1 Neuroimmunology and Neurochemistry Research Group, Department of Psychiatry and Psychotherapy, Medical Center-University of Freiburg, Faculty of Medicine, University of Freiburg, 79104 Freiburg, Germany; lusun.jane@gmail.com (L.S.); matthias.apweiler@uniklinik-freiburg.de (M.A.); rischulzki@gmail.com (R.S.); 2 Department of Psychiatry and Psychotherapy, Medical Center-University of Freiburg, Faculty of Medicine, University of Freiburg, 79104 Freiburg, Germany; claus.normann@uniklinik-freiburg.de; 3 Institute of Biological and Chemical Systems-Functional Molecular Systems (IBCS-FMS), Karlsruhe Institute of Technology (KIT), Kaiserstrasse 12, 76131 Karlsruhe, Germany; christoph.grathwol@kit.edu (C.W.G.); braese@kit.edu (S.B.); 4 Institute of Organic Chemistry, Karlsruhe Institute of Technology (KIT), 76131 Karlsruhe, Germany

**Keywords:** neuroinflammation, IL-1β, SK-N-SH, cardenolides, digitoxigenin, digitoxigenone, coroglaucigenin

## Abstract

Neuroinflammation is a key feature of neurological disorders such as Alzheimer’s or Parkinson’s disease, depression or amyotrophic lateral sclerosis. This study investigates the anti-neuroinflammatory effects of three cardenolide derivatives, digitoxigenin, digitoxigenone, and coroglaucigenin (all at concentrations of 1, 5, 10, and 25 µM), in IL-1β-stimulated human neuroblastoma SK-N-SH cells. All compounds significantly reduced PGE_2_ production without affecting cell viability by downregulation of cyclooxygenase (COX)-2 and microsomal prostaglandin E synthases (mPGES)-1 expression and synthesis. Among them, digitoxigenin notably inhibited phosphorylation of p38 mitogen-activated protein kinase (MAPK) and nuclear factor-kappa B (NF-κB) and suppressed interleukin (IL)-6 and IL-8 expression and release. These findings demonstrate that cardenolide derivatives exert potent anti-neuroinflammatory effects by at least partially targeting key enzymes of the arachidonic acid pathway and modulating MAPK/NF-κB signaling, highlighting their potential as novel therapeutic candidates for neuroinflammatory diseases. Future research should focus on effects of the cardenolides in mixed-cell cultures, organoids and in vivo experiments, to evaluate possible side effects and the potential in disease models.

## 1. Introduction

A substantial body of research emphasizes the critical role of neuroinflammation in neurological and psychiatric diseases, such as Alzheimer’s Disease (AD), Parkinson’s Disease (PD), amyotrophic lateral sclerosis (ALS) and depression [1,2]. In light of this, pharmacological interventions focusing on neuroinflammation could present new therapeutic prospects for the mentioned diseases. A wide variety of inflammatory mediators contribute to the mechanisms involved in neuroinflammation.

Arachidonic acid (AA) is a polyunsaturated fatty acid, released from the cell membrane’s phospholipids under the enzymatic action of the cytosolic phospholipase A_2_ (cPLA_2_) during inflammatory action and acts as precursor to pro-inflammatory eicosanoids [3]. AA regulates membrane fluidity, influences the function of relevant membrane proteins, participates in cellular signaling, and plays a vital role in maintaining cell and organelle integrity as well as vascular permeability. AA, serving as a substrate for various pathways, undergoes metabolization to different types of pro-inflammatory lipid mediators, including prostaglandins (PGs) and thromboxanes (TXAs) through the cyclooxygenase (COX) pathway, and leukotrienes (LTs) through the lipoxygenase (LOX) pathway [4]. AA can be converted to prostaglandin H_2_ (PGH_2_) by cyclooxygenases, which divide into two isoforms (COX-1 and COX-2) [5]. Then, PGH_2_ is further converted to PGE_2_ by microsomal prostaglandin E synthases-1 and -2 (mPGES-1 and mPGES-2) as well as cytosolic prostaglandin E synthase (cPGES). It is reported that pro-inflammatory cytokines and COX-2 derived PGE_2_ may play a vital role in seizure generation and exacerbation [6]. Furthermore, AA, which acts as a second messenger in the G-protein coupled receptor (GPCR)-signaling cascade, plays an important role in CCL2-induced chemotaxis [7]. Preliminary reports showed that AA is responsible for the increase in pro-inflammatory monocyte chemoattractant protein 1 (MCP-1, synonym to CCL2), interleukin (IL)-6 release as well as the upregulation of COX-2 and toll-like receptor 2 (TLR2) expression [8]. It has been revealed that triggering of chemotaxis by CCL2 is completely inhibited by the antisense knockout of cPLA_2_ [9].

Cardenolides are a group of steroid-derived natural products that can bind to Na^+^/K^+^-ATPase with an inhibiting activity and traditionally were used to treat congestive heart failure and arrhythmias [10]. Recent research indicates that cardenolides may also possess anti-inflammatory properties. For example, cardenolides can decrease the production of reactive oxygen species (ROS), which are often involved in the propagation of inflammation by inducing oxidative stress [11]. It is reported that cardenolides can reduce the production of various inflammatory mediators, including nitric oxide (NO) and PGs, by inhibiting the activity of enzymes like inducible nitric oxide synthase (iNOS) and COX-2. Furthermore, cardenolides inhibit the nuclear factor-kappa B (NF-κB) signaling pathway regulating inflammation [12]. While the primary use of cardenolides currently remains in the treatment of heart conditions, their anti-inflammatory properties suggest potential therapeutic applications in diseases characterized by chronic inflammation. Further research is needed to fully understand the mechanisms underlying these effects and to explore their potential in clinical settings for treating inflammatory diseases.

The regulation of pro-inflammatory mediators and involved enzymes is controlled by different intracellular signaling pathways, such as NF-κB and mitogen-activated protein kinases (MAPK) dependent pathways [13,14]. NF-κB is a transcription factor considered to be the critical central regulator of the inflammatory process [15]. The transcription factor regulates gene expressions of the pro-inflammatory cytokines (IL-6, tumor necrosis factor (TNF)α, TNFβ, IL-2 etc.), chemokines (IL-8, CCL-2, CCL-5, etc.), adhesion molecules, inducible enzymes (COX-2 and iNOS), growth factors, some acute phase proteins and immune receptors [16,17]. NF-κB has therefore become a critical molecular target in drug discovery, and several natural and synthetic compounds are currently being investigated for their potential to inhibit NF-κB [18,19]. Additionally, NF-κB is involved in the IL-1β-induced activation of cPLA_2_ and the associated inflammatory pathways [20,21].

Therefore, considering the central role of the AA/COX-2/PGE_2_ pathway and inflammatory signaling cascades such as NF-κB and MAPK in neuroinflammation, and given the emerging evidence of cardenolides exerting anti-inflammatory effects, it is plausible to hypothesize that cardenolide derivatives may attenuate neuroinflammatory responses. However, the potential of cardenolides in the context of neuroinflammation remains largely unexplored. In this study, we aimed to investigate the anti-neuroinflammatory effects of three cardenolide derivatives—digitoxigenin, digitoxigenone, and coroglaucigenin—in an in vitro model of IL-1β-activated human SK-N-SH neuroblastoma cells, focusing on their impact on PGE_2_ production, modulation of expression and translation of key enzymes of the AA cascade (COX-2 and mPGES-1), pro-inflammatory cytokines (IL-6 and IL-8), and underlying signaling pathways.

## 2. Results

### 2.1. Effects of Digitoxigenin, Digitoxigenone and Coroglaucigenin on Cell Viability

**Figure 1:**
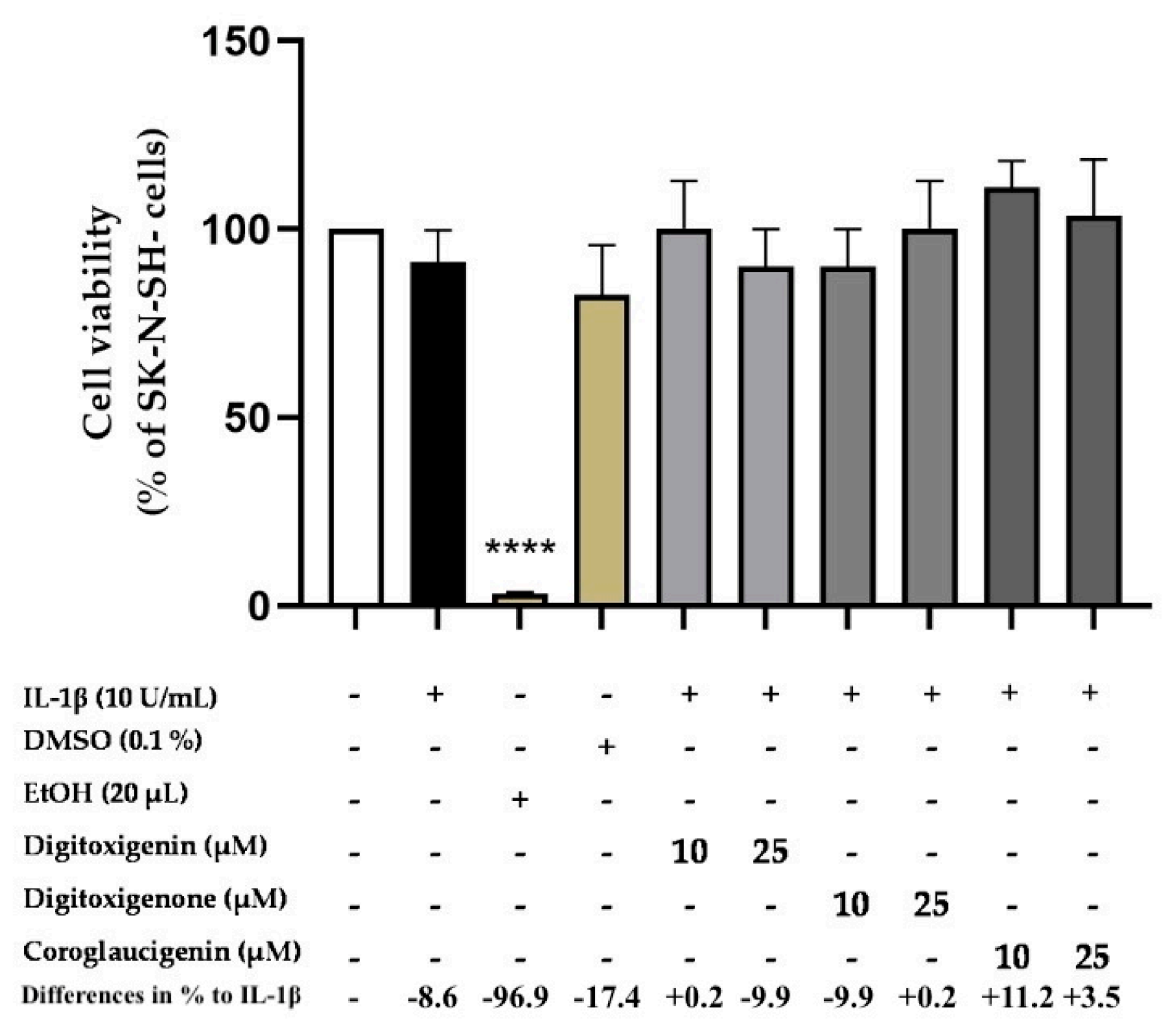
Effects of digitoxigenin (light grey), digitoxigenone (mid grey) and coroglaucigenin (dark grey) on cell viability. Cell viability was measured after 24 h of treatment by color change due to MTT reduction. Values are presented as mean ± SD of four independent experiments. Statistical analysis was performed using one-way ANOVA with Dunnett’s post hoc test with **** *p* < 0.0001 compared to untreated cells.

To investigate possible cytotoxic effects of digitoxigenin, digitoxigenone and coroglaucigenin (10 and 25 μM) in SK-N-SH cells, an MTT assay was performed. As illustrated in Figure 1, digitoxigenin, digitoxigenone and coroglaucigenin showed no significant cytotoxicity in IL-1β-stimulated SK-N-SH cells. The solvent DMSO at 0.1% concentration had no significant impact on cell viability at the same time. In contrast, 20% ethanol, used as a positive control, significantly induced cell death. Therefore, further experiments were performed with those three compounds at concentrations up to 25 μM.

### 2.2. Digitoxigenin, Digitoxigenone and Coroglaucigenin Significantly Reduce IL-1β-Induced PGE_2_ Release

**Figure 2:**
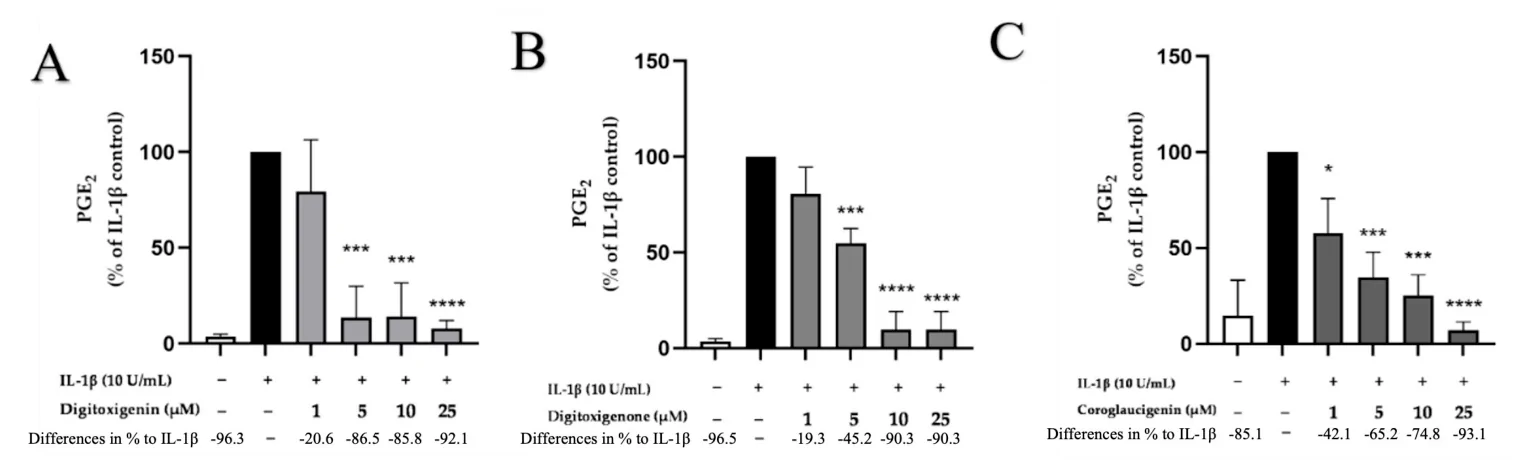
Effects of digitoxigenin (**A**), digitoxigenone (**B**) and coroglaucigenin (**C**) on the release of PGE_2_ in IL-1β-stimulated SK-N-SH cells. Cells were stimulated as described before. Values are presented as the mean ± SD of three independent experiments. Statistical analysis was performed using one-way ANOVA with Dunnett’s post hoc tests with * *p* < 0.05, *** *p* < 0.001 and **** *p* < 0.0001 compared to IL-1β.

To investigate whether the compounds exert anti-inflammatory effects, SK-N-SH cells were pre-incubated with digitoxigenin, digitoxigenone and coroglaucigenin (1, 5, 10, and 25 μM) for half an hour and then stimulated with or without IL-1β (10 U/mL) for the next 24 h. A significant increase in the production of PGE_2_ in IL-1β-treated cells was observed. As shown if Figure 2A–C, pretreatment with digitoxigenin, digitoxigenone and coroglaucigenin revealed a concentration-dependent decrease of PGE_2_ release. Digitoxigenin showed a significant inhibition of PGE_2_ production starting at concentrations of 5 µM (Figure 2A). Digitoxigenone also exerted a concentration-dependent inhibition of IL-1β-induced PGE_2_ production reaching statistical significance starting at 5 µM (Figure 2B), but less potent at 5 µM when compared to digitoxigenin. Coroglaucigenin strongly and significantly inhibited PGE_2_ synthesis in a concentration-dependent manner reaching basal levels in its highest concentration of 25 µM. In comparison to digitoxigenin and digitoxigenone, coroglaucigenin showed the strongest effects at 1 µM (Figure 2C).

### 2.3. Digitoxigenin, Digitoxigenone and Coroglaucigenin Significantly Reduce IL-1β-Induced COX-2 Expression and Synthesis

**Figure 3:**
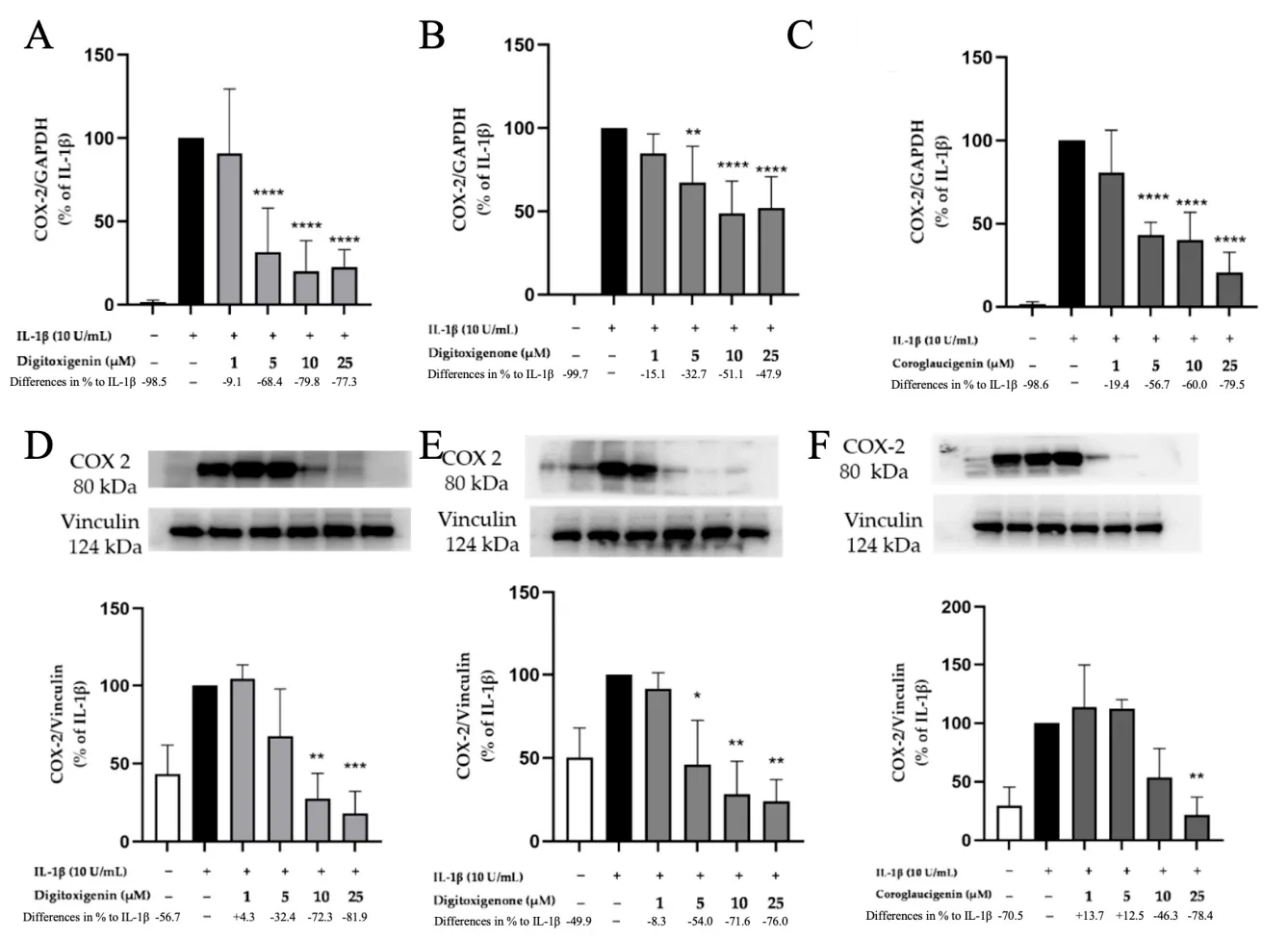
Effects of Digitoxigenin (**A**,**D**), Digitoxigenone (**B**,**E**) and Coroglaucigenin (**C**,**F**) on the expression (**A**–**C**) and synthesis (**D**–**F**) of COX-2 in IL-1β-stimulated SK-N-SH cells. Cells were stimulated as described before. Values are presented as the mean ± SD of three independent experiments. Statistical analysis was performed using one-way ANOVA with Dunnett’s post hoc tests with * *p* < 0.05, ** *p* < 0.01, *** *p* < 0.001 and **** *p* < 0.0001 compared to IL-1β.

Since a strong reduction of PGE_2_ release was found, the main enzymes of the AA/COX/PGE_2_ pathway were focused in the next steps. First, the effects of digitoxigenin, digitoxigenone and coroglaucigenin on *COX-2* expression were investigated using qPCR, which revealed that all three compounds significantly reduced IL-1β-induced *COX-2* expression in a dose-dependent manner (Figure 3A–C). A clear reduction in *COX-2* transcription was already evident at 5 µM for all compounds; Digitoxigenin and coroglaucigenin showed a stronger reduction of *COX-2* expression at 25 µM than digitoxigenone. Consequently, it was determined whether the effects of digitoxigenin, digitoxigenone and Coroglaucigenin on PGE_2_ release were mediated through an inhibition of the synthesis of those enzymes. As shown in Figure 3D–F, all three cardenolides attenuated COX-2 synthesis in a concentration-dependent manner, though with different potencies. Digitoxigenin significantly reduced COX-2 synthesis starting at concentrations of 10 µM to basal levels (Figure 3D). Digitoxigenone also decreased synthesis of COX-2 beyond basal levels starting at 5 µM (Figure 3E). Furthermore, coroglaucigenin only exerted significant inhibition of COX-2 synthesis at the concentration of 25 µM (Figure 3F), but again to basal levels.

### 2.4. Digitoxigenin, Digitoxigenone and Coroglaucigenin Suppress PGE_2_ Production by Inhibiting mPGES-1 Expression and Synthesis in IL-1β Activated SK-N-SH Cells

**Figure 4:**
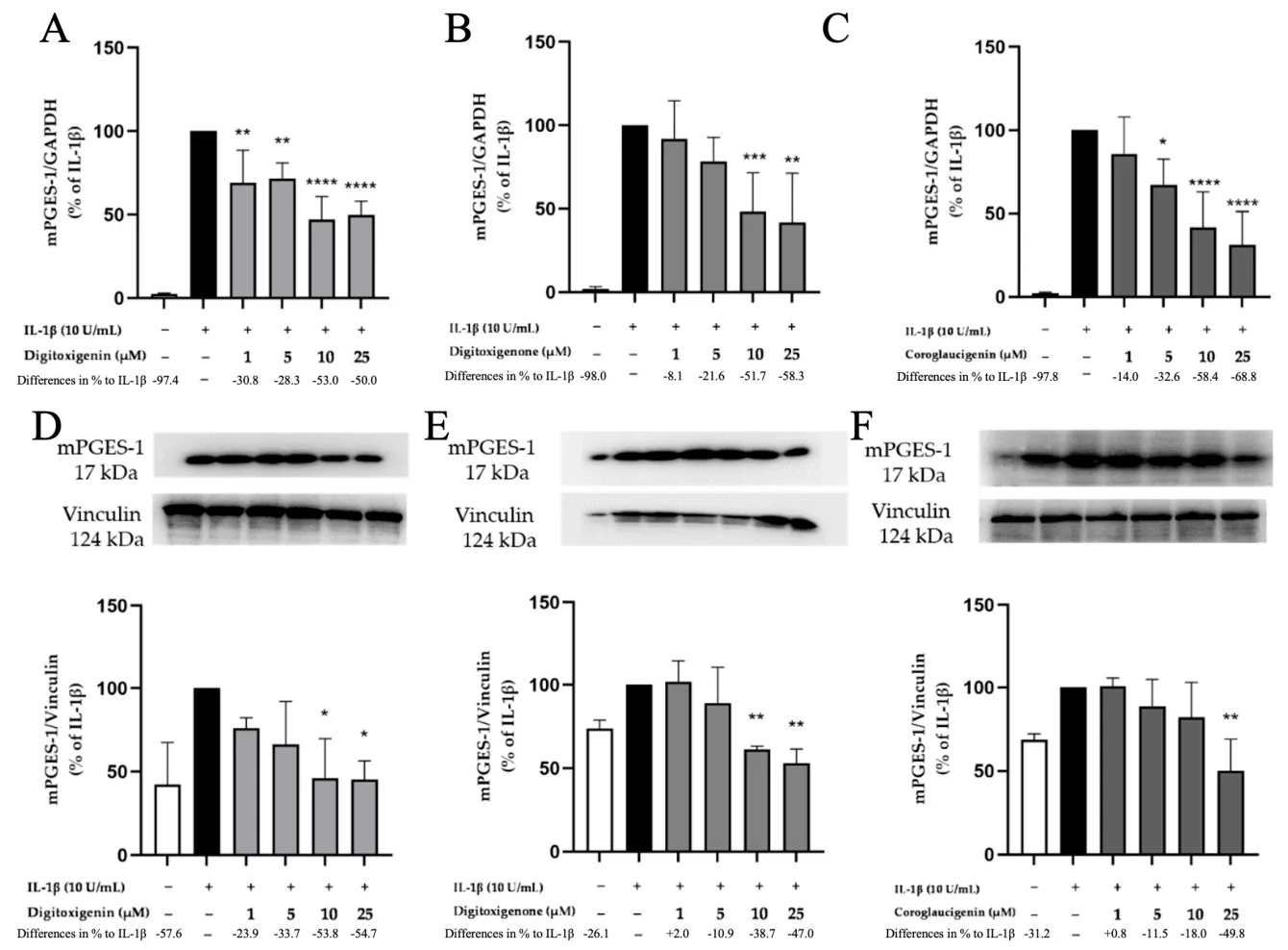
Effects of digitoxigenin (**A**,**D**), digitoxigenone (**B**,**E**) and coroglaucigenin (**C**,**F**) on mPGES-1 expression (**A**–**C**) and synthesis (**D**–**F**) in IL-1β-stimulated SK-N-SH cells. Cells were stimulated as described before. Values are presented as the mean ± SD of three independent experiments. Statistical analysis was performed using one-way ANOVA with Dunnett’s post hoc tests with * *p* < 0.05, ** *p* < 0.01, *** *p* < 0.001 and **** *p* < 0.0001 compared to IL-1β.

PGE_2_ is synthesized through the enzymatic action of COX-2 and mPGES-1. Consequently, it was determined whether the effect of digitoxigenone and coroglaucigenin on PGE_2_ was mediated through an inhibition of the synthesis of those enzymes. As shown in Figure 4, *mPGES-1* expression was measured by qPCR following IL-1β stimulation (4 h). Pretreatment with digitoxigenin, digitoxigenone and coroglaucigenin markedly and dose-dependently suppressed IL-1β-induced *mPGES-1* expression (Figure 4A–C). Digitoxigenin, digitoxigenone and coroglaucigenin exhibited significant reduction starting at the concentrations of 1 µM, 10 µM and 5 µM, respectively. All three cardenolides attenuated mPGES-1 synthesis in a concentration-dependent manner, though with different potencies. Digitoxigenin and digitoxigenone both significantly decreased synthesis of mPGES-1 starting at 10 µM (Figure 4D,E). Furthermore, coroglaucigenin only exerted significant inhibition of mPGES-1 at the concentration of 25 µM (Figure 4F).

### 2.5. Effects of Digitoxigenin, Digitoxigenone and Coroglaucigenin on COX-Activity

**Figure 5:**
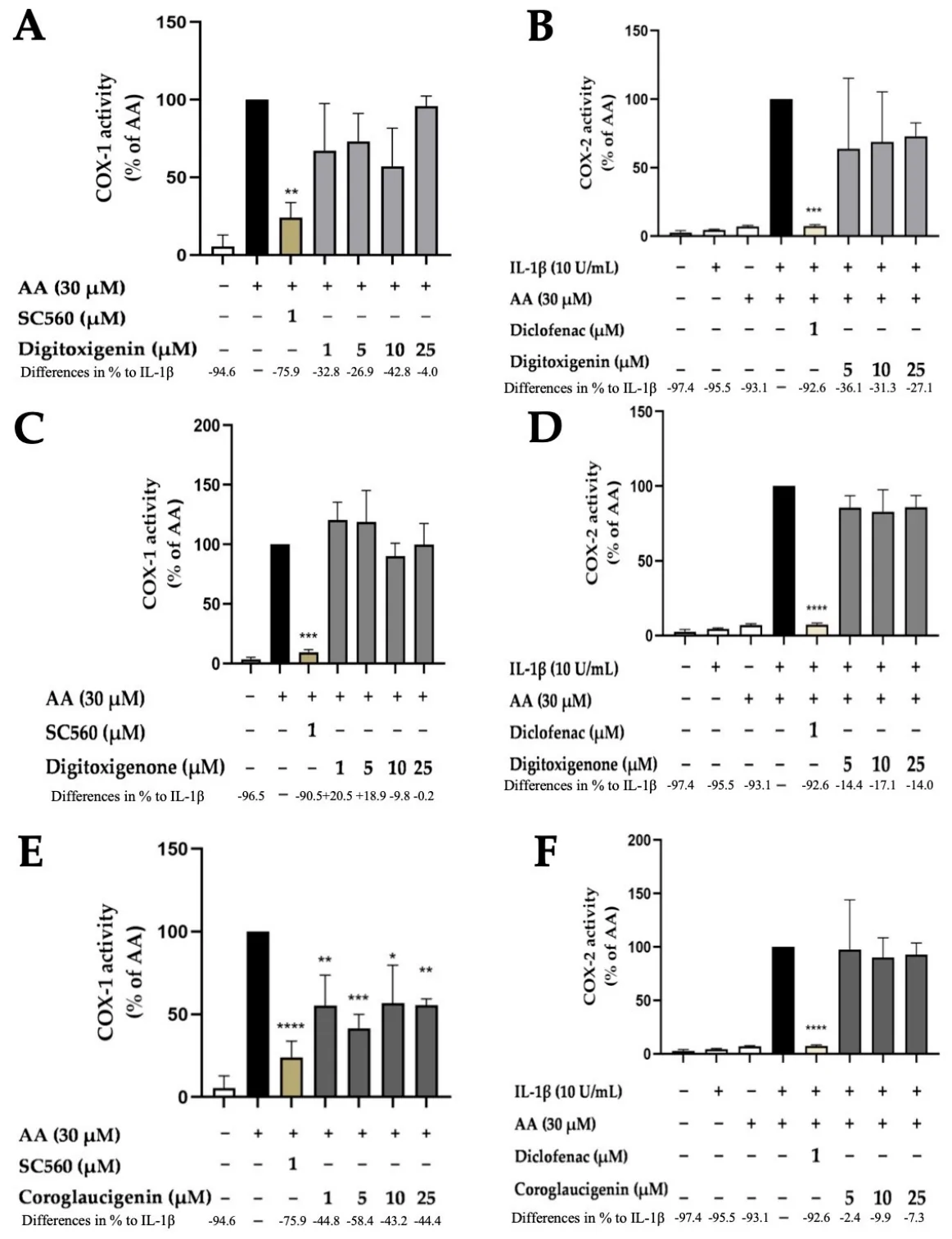
Effects of digitoxigenin (**A**,**B**), digitoxigenone (**C**,**D**) and coroglaucigenin (**E**,**F**) on COX-1 (**A**,**C**,**E**) and COX-2 (**B**,**D**,**F**) enzymatic activity in IL-1β-stimulated SK-N-SH cells. Cells were stimulated as described before. Values are presented as the mean ± SD of three independent experiments. Statistical analysis was performed using one-way ANOVA with Dunnett’s post hoc tests with * *p* < 0.05, ** *p* < 0.01, *** *p* < 0.001 and **** *p* < 0.0001 compared to IL-1β.

Besides COX synthesis and expression, enzyme activities of COX-1 and COX-2 were examined as another possible mechanism of the observed PGE_2_ reduction independent of COX-2 synthesis or expression. As expected, AA significantly enhanced the measured PGE_2_ production, since it is the substrate for the COX/mPGES/PGE_2_ pathway. Since COX-2 is induced by inflammatory stimuli and is dependent on its substrate AA, the highest COX-2 activity is observed in IL-1β and AA treated cells. The selective COX-1 inhibitor SC-560 potently decreased COX-1 activity, while the COX-1 and COX-2 inhibitor diclofenac in concentration of 1 µM significantly inhibited COX-2 activity. Neither digitoxigenin nor digitoxigenone affected COX-1 or COX-2 activity in concentrations between 1 µM and 25 µM (Figure 5A–D). Coroglaucigenin, in contrast, significantly inhibited COX-1 activity to around 60% (Figure 5E). Interestingly, the concentration of 5 µM showed the strongest reduction. No effects on COX-2 activity were observed for coroglaucigenin (Figure 5F).

### 2.6. Effects of Digitoxigenin, Digitoxigenone and Coroglaucigenin on Phosphorylation of p38 MAPK and NF-κB

**Figure 6:**
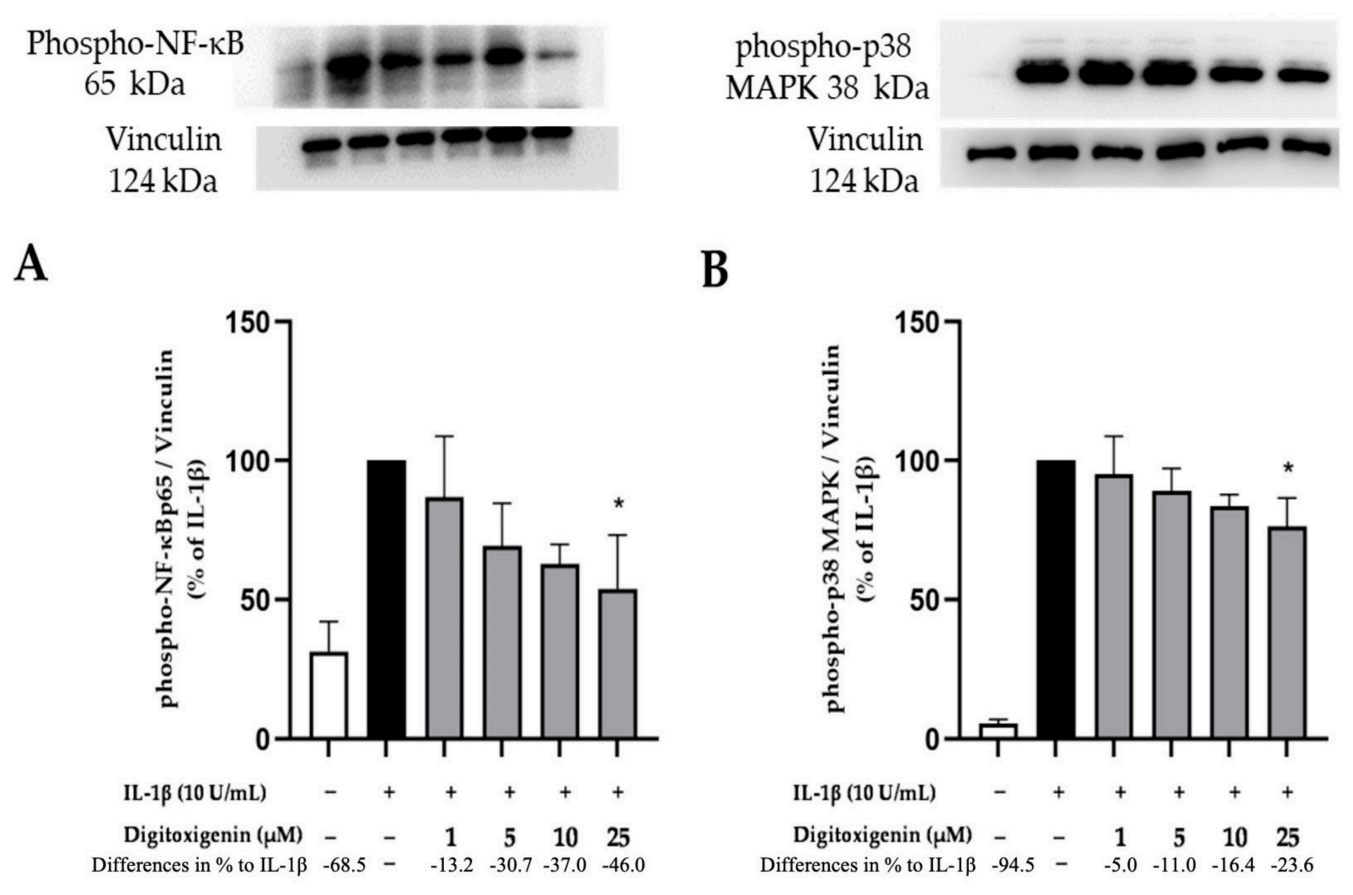
Effects of digitoxigenin on phosphorylation of NF-κB (**A**) and p38 MAPK (**B**) in IL-1β-stimulated SK-N-SH cells. Cells were stimulated as described before. Values are presented as the mean ± SD of three independent experiments. Statistical analysis was performed using one-way ANOVA with Dunnett’s post hoc tests with * *p* < 0.05 compared to IL-1β.

The MAPK signaling pathway, consisting of SAPK/JNK, p38 MAPK and ERK1/2, is integral to the regulation of various cellular activities. NF-κB, a family of dimeric transcription factors, plays a crucial role in orchestrating inflammatory responses [22]. Therefore, effects of digitoxigenin, digitoxigenone and coroglaucigenin on the phosphorylation of p38 MAPK and NF-κB in SK-N-SH cells were investigated. Figure 6 shows that digitoxigenin exerted a significant concentration-dependent inhibition of p38 MAPK and NF-κB phosphorylation reaching significance at a concentration of 25 µM. Basal phosphorylation levels were not achieved in the highest concentration. However, digitoxigenone and coroglaucigenin did not significantly alter the phosphorylation of p38 MAPK and NF-κB (Supplementary Figure S1).

### 2.7. Effects of Digitoxigenin, Digitoxigenone and Coroglaucigenin on IL-6 Release and Expression

**Figure 7:**
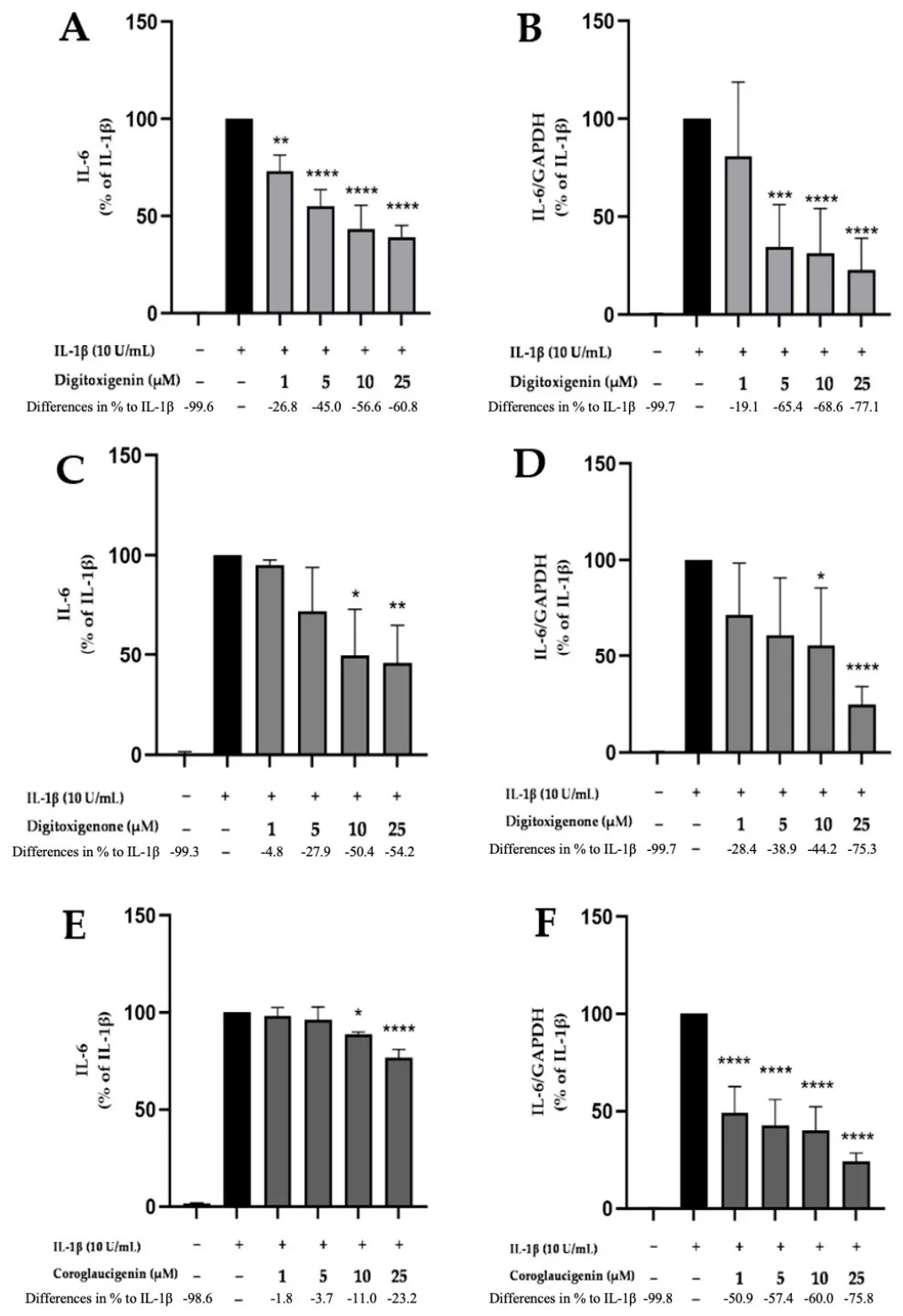
Effects of digitoxigenin (**A**,**B**), digitoxigenone (**C**,**D**) and coroglaucigenin (**E**,**F**) on the expression (**B**,**D**,**F**) and release (**A**,**C**,**E**) of IL-6 in IL-1β-stimulated SK-N-SH cells. Cells were stimulated as described before. Values are presented as the mean ± SD of three independent experiments. Statistical analysis was performed using one-way ANOVA with Dunnett’s post hoc tests with * *p* < 0.05, ** *p* < 0.01, *** *p* < 0.001 and **** *p* < 0.0001 compared to IL-1β.

The cytokine IL-6 is a prototypical cytokine for maintaining homeostasis and vital mediator of the inflammatory process [23]. The expression and release of IL-6 in IL-1β-treated SK-N-SH cells was measured. SK-N-SH cells were pretreated with digitoxigenin, digitoxigenone and coroglaucigenin (1, 5, 10, and 25 µM) for 30 min. Afterwards, cells were incubated with IL-1β for 4 h (qPCR) or 24 h (ELISA). Expression and synthesis of IL-6 were both strongly induced by IL-1β stimulation. Pretreatment with digitoxigenin, digitoxigenone and coroglaucigenin showed a comparable significant and concentration-dependent reduction of IL-6 expression and release, though the potency profile differed among the compounds (Figure 7). Notably, digitoxigenin was the most potent compound, reaching a significant reduction of IL-6 release starting at 1 µM (Figure 7A), while digitoxigenone and coroglaucigenin required a higher concentration of 10 µM (Figure 7C,E). In contrast, coroglaucigenin was the most potent inhibitor of IL-6 expression starting at the concentration of 1 µM (Figure 7F), digitoxigenin and digitoxigenone exert the inhibition from 5 µM (Figure 7B) and 10 µM (Figure 7D), each.

### 2.8. Effects of Digitoxigenin, Digitoxigenone and Coroglaucigenin on IL-8 Release and Expression

**Figure 8:**
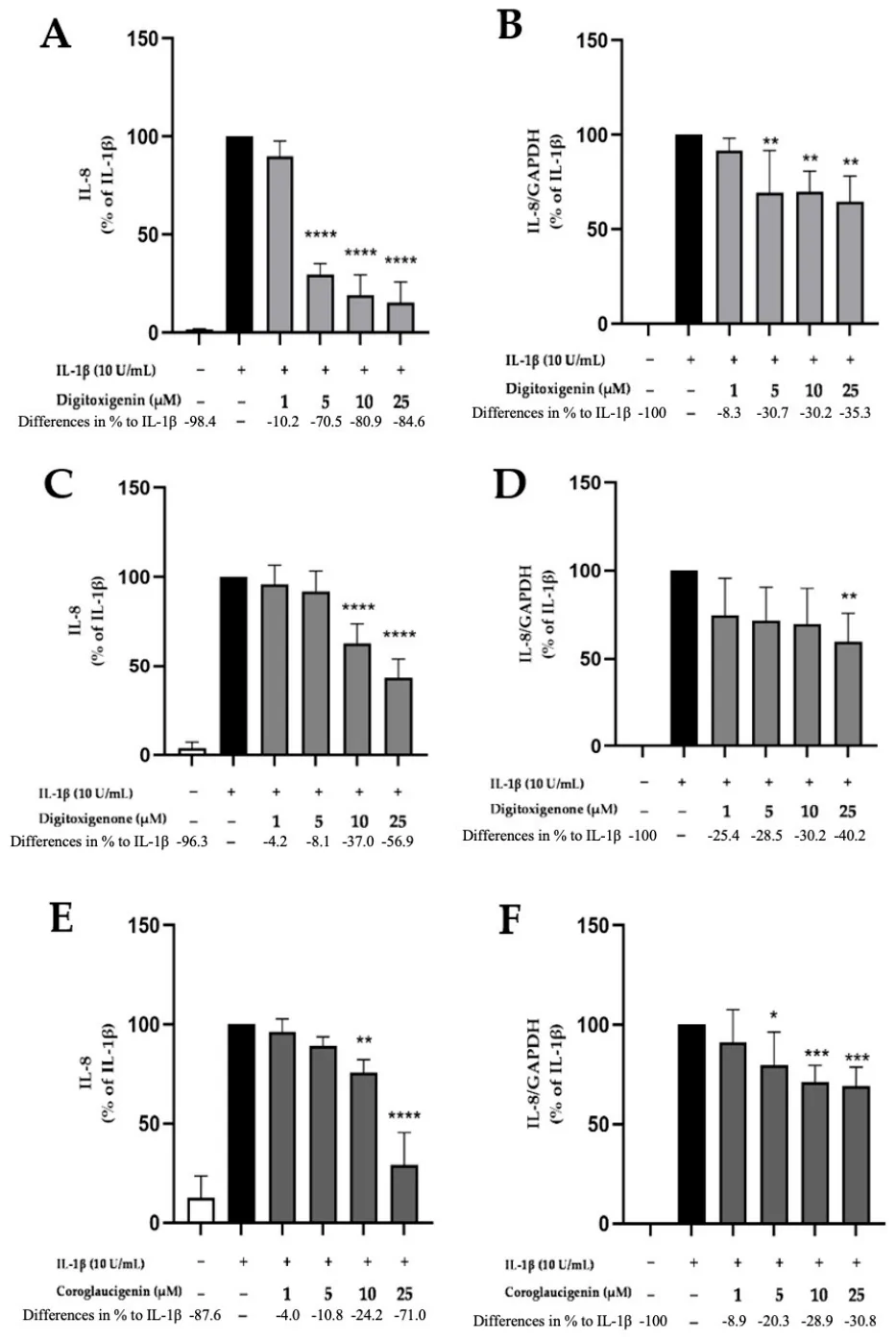
Effects of digitoxigenin (**A**,**B**), digitoxigenone (**C**,**D**) and coroglaucigenin (**E**,**F**) on the expression (**B**,**D**,**F**) and release (**A**,**C**,**E**) of IL-8 in IL-1β-stimulated SK-N-SH cells. Cells were stimulated as described before. Values are presented as the mean ± SD of three independent experiments. Statistical analysis was performed using one-way ANOVA with Dunnett’s post hoc tests with * *p* < 0.05, ** *p* < 0.01, *** *p* < 0.001 and **** *p* < 0.0001 compared to IL-1β.

IL-8 is a pro-inflammatory cytokine, which directs migration of cells to a site of inflammation [24]. The stimulation of SK-N-SH cells with IL-1β (10 U/mL) for 4 h (qPCR) or 24 h (ELISA) caused a significant increase in the expression and synthesis of IL-8, respectively, as shown in Figure 8. Digitoxigenin most potently inhibited IL-8 release, with significant reduction evident at 5 µM (Figure 8A). Digitoxigenone and coroglaucigenin were less potent, whereas both required a concentration of 10 μM to elicit a statistically significant reduction to baseline levels (Figure 8C,E).

Pretreatment with digitoxigenin and coroglaucigenin led to the strong inhibition of *IL-8* expression starting at concentrations of 5 µM (Figure 8B,F); however, digitoxigenone only exerted significant reduction of *IL-8* expression at the concentration of 25 µM (Figure 8D).

## 3. Discussion

In this study, we investigated anti-inflammatory effects of the cardenolides digitoxigenin, digitoxigenone and coroglaucigenin in IL-1β-activated SK-N-SH cells. All compounds demonstrated anti-neuroinflammatory effects by inhibiting IL-1β-induced PGE_2_ release via inhibition of mPGES-1 and COX-2 expression and synthesis. None of the compounds inhibited the COX-2 enzymatic activity and only coroglaucigenin inhibited COX-1 activity. Furthermore, the investigations showed that digitoxigenin, digitoxigenone and coroglaucigenin additionally reduced the pro-inflammatory cytokines IL-6 and IL-8. In addition, the underlying signaling mechanisms might be partially dependent on the p38 MAPK signaling pathway and NF-κB signaling at least for digitoxigenin.

Cardenolides are known for their narrow therapeutic ranges due to their cytotoxicity. However, in the current study we did not observe a significant cytotoxicity in the used SK-N-SH cells. Other studies show that differences in the sensitivity to cytotoxic effects of cardenolides in different cells might be explained by differences in the specific sodium pump subunit expression [25]. Due to the cell-specific ratios of Na^+^/K^+^-ATPase subunits, findings for cardenolide-induced cytotoxicity vary between different cell lines. Furthermore, aglycone forms of cardenolides such as digitoxigenin generally exert less cytotoxic effects compared to glycosylated counterparts digitoxin or digoxin [26,27].

Anti-neuroinflammatory effects of cardenolides are increasingly investigated in the central nervous system (CNS). In a recent study, the anti-inflammatory and neuroprotective effects of cardenolides were demonstrated using both Aβ_1–42_-stimulated BV-2 microglial cultures and FAD4T transgenic mouse models of AD. Ouabain attenuated Aβ-induced microglial activation by upregulating TREM2 and activating the PI3K/AKT signaling pathway, thereby reducing pro-inflammatory cytokines such as IL-1β and TNF-α, promoting M2-type polarization, and ultimately mitigating neuroinflammation and neuronal damage [28]. In another study, cardenolides demonstrated protective effects in inflammatory bowel disease (IBD), primarily through modulation of inflammatory signaling and inhibition of immune cell activation [29]. Studies in rodents with colitis or arthritis have shown that cardenolides decrease *COX-2* and *iNOS* expression, reduce leukocyte infiltration, and inhibit the release of reactive oxygen species (ROS), thereby mitigating tissue damage [30]. Furthermore, indirect evidence suggests the potential effects of cardenolides in treating chronic inflammatory conditions such as cardiovascular diseases through their ability to modulate Na^+^/K^+^-ATPase signaling, reduce oxidative stress, and suppress inflammasome activation [31].

Digitoxigenin, digitoxigenone and coroglaucigenin are structurally closely related to one another: Digitoxigenone is derived directly from digitoxigenin through oxidation at C-3, replacing the 3β-hydroxyl group with a 3-oxo group, whereas coroglaucigenin retains the 3β-hydroxy group but carries an additional hydroxyl group at C19. All three compounds inhibited the AA/COX-2/PGE_2_ pathway on different levels of the enzymes involved, leading to a strong reduction of PGE_2_ in IL-1β-treated SK-N-SH cells and upstream members of the arachidonic acid pathway (COX-2/mPGES-1). For most parameters, the three compounds show a broadly similar biological activity profile, with Digitoxigenin exhibiting slightly superior efficacy compared to the 3-oxo and the 19-hydroxy derivatives digitoxigenone and coroglaucigenin, respectively. One could hypothesize that digitoxigenin might show the optimal balance between polarity and lipophilicity. The less polar digitoxigenone is likely less aqueously soluble and coroglaucigenin might exert less diffusion over cell membranes due to the higher polarity. For that reason, digitoxigenin might reach higher intracellular concentrations. Another explanation for the differences in the biological activity might be the additional C19 hydroxyl group of coroglaucigenin that might interfere with a potential hydrophobic binding pocket of unknown targets. Compared to digitoxigenone, digitoxigenin and coroglaucigenin are furthermore hydrogen-bond donors at C3, which might be relevant for their biological activity.

In line with the PGE_2_ reduction, a decreased expression and synthesis of COX-2 and mPGES-1 was demonstrated, which likely accounts for the inhibition of PGE_2_ synthesis. Even if the three compounds did not reduce those enzymes to baseline levels of untreated cells, the synergistic effect of the inhibition on different cascade levels explains the strong decrease of PGE_2_ release observed. In other studies, cardenolides, such as Ouabain, downregulated COX-2 expression and inhibited PGE_2_ production, thereby suppressing inflammatory responses in various models, including LPS-induced neuroinflammation in rat hippocampus [32] and carrageenan/zymosan-induced peripheral inflammation in mice [33]. The suppression of the AA/COX-2/PGE_2_ pathway is particularly relevant in the context of neuroinflammation, as excessive PGE_2_ production has been implicated in the pathogenesis of neurodegenerative diseases such as AD, PD, and multiple sclerosis. PGE_2_ is known to mediate fever, pain, blood brain barrier disruption, and injury [34,35,36]. Sustained activation of the COX-2/mPGES-1 axis has been shown to drive chronic inflammation and is associated with increased amyloid-β deposition and tau phosphorylation in AD, as well as dopaminergic neuron degeneration in PD models [37,38]. Thus, targeting this pathway could offer therapeutic benefits by dampening inflammatory signaling and preserving neuronal integrity. Given that mPGES-1 functions downstream of COX-2 and is inducible during inflammation, dual inhibition of both enzymes represents a more effective strategy than COX-2 blockade alone, potentially avoiding some of the adverse effects seen with nonselective COX inhibitors [37]. However, since coroglaucigenin exhibits COX-1 inhibitory activity, careful consideration of selectivity and safety margins is required during drug development. Interestingly, COX-1 inhibition appears to be structurally linked to the additional C19 hydroxyl group, which is absent in digitoxigenin and digitoxigenone. Traditional nonselective NSAIDs that inhibit COX-1 are known to cause gastrointestinal ulceration and bleeding due to the loss of prostaglandin-mediated mucosal protection [39]. Avoiding hydroxylation at C19, therefore, could serve as a structural design principle for developing cardenolide derivatives that leave COX-1 activity unaffected, lowering the risk of such gastrointestinal side effects. Our findings underscore the therapeutic promise of cardenolide derivatives as modulators of this pathway, particularly in conditions where COX-2 and mPGES-1 are pathologically upregulated, such as AD, PD, multiple sclerosis, and ALS. In these neurodegenerative disorders, chronic neuroinflammation is accompanied by excessive activation of the arachidonic acid cascade, resulting in persistent COX-2 and mPGES-1 induction and subsequent overproduction of PGE_2_, which contributes to neuronal injury and disease progression [40,41].

Consistent with previous studies, our results suggest that the anti-neuroinflammatory effects of cardenolides may be partly mediated through suppression of key inflammatory signaling pathways, particularly the MAPK family. As demonstrated in LPS-activated microglia, the phosphorylation levels of p38 MAPK, ERK1/2, and SAPK/JNK are significantly upregulated during neuroinflammatory responses, driving *COX-2* and pro-inflammatory cytokine expression [42]. Similarly, in our IL-1β-induced SK-N-SH cell model, digitoxigenin treatment attenuated the activation of p38 MAPK, which are known to regulate *COX-2* and *mPGES-1* expression [43]. It is crucial to note that this inhibition was only observed for at the highest concentration (25 µM), while digitoxigenone and coroglaucigenin did not significantly alter the phosphorylation of p38 MAPK or NF-κB. This key observation indicates that the potent suppression of PGE_2_, COX-2, mPGES-1, IL-6, and IL-8 by digitoxigenone and coroglaucigenin is largely independent of the canonical p38 MAPK and NF-κB pathways investigated here. Interestingly, although the NF-κB pathway plays a crucial role in inflammation by promoting transcription of cytokines like TNF-α and IL-6, our findings are consistent with previous research showing that certain anti-inflammatory agents such as rice bran extract selectively affect MAPK pathways without significantly altering NF-κB activation, as evidenced by the lack of change in IκB-α degradation [42,44]. The disparate signaling effects among the three cardenolide derivatives suggest that they may engage with distinct upstream targets. For digitoxigenin, the anti-inflammatory outcome may indeed involve the noted attenuation of phosphorylation of p38 MAPK and NF-κB at high doses. In contrast, for digitoxigenone and coroglaucigenin, the effects are likely mediated through alternative up- or downstream kinases or signaling nodes that converge directly on the transcriptional or post-transcriptional regulation of key inflammatory enzymes like COX-2 and mPGES-1, bypassing the pathways investigated in this study. Moreover, a recent study demonstrated that 3′,4′,5,7-tetramethoxyflavone suppresses neuroinflammation via concurrent inhibition of both MAPK and NF-κB signaling in LPS-induced microglia [45]. This indicates that cardenolides, depending on the specific compound, may exhibit a spectrum of signaling modulation, with some members like digitoxigenin potentially influencing MAPK-mediated pathways, while others operate through distinct, yet-to-be-identified mechanisms to achieve a convergent anti-inflammatory outcome. Other cardenolides have been investigated regarding their anti-inflammatory properties as well. Digoxin reduces pro-inflammatory cytokines, such as IL-6 and TNF-α in a collagen-induced murine arthritis model [46], as it is shown for digitoxin via inhibition of NF-κB signaling in endothelial cells, leading to reduction of CCL-2 [47] and possibly other cytokines. However, the glycosylated cardenolides show higher cytotoxicity and therefore have a smaller therapeutic range. The stronger affinity to the Na^+^/K^+^-ATPase might lead to higher anti-inflammatory capacity as well, so future studies should compare glycosylated and aglycone cardenolides in the same model.

These findings highlight the emerging role of cardenolides as promising candidates for anti-inflammatory drug development, although further research is needed to fully elucidate their mechanisms and therapeutic applications. While promising, the dose-dependent toxicity of cardenolides remains a concern, as digoxin becomes arrhythmogenic above ~1.7 ng/mL, digitoxin shows a small clinical range (~20–33 nM), and ouabain shows cytotoxicity above ~7.5 nM [48,49]. Future studies should aim to delineate the precise molecular targets of cardenolides in the CNS and explore their potential therapeutic applications for neuroinflammatory conditions.

## 4. Materials and Methods

### 4.1. Cardenolides

**Figure 9:**
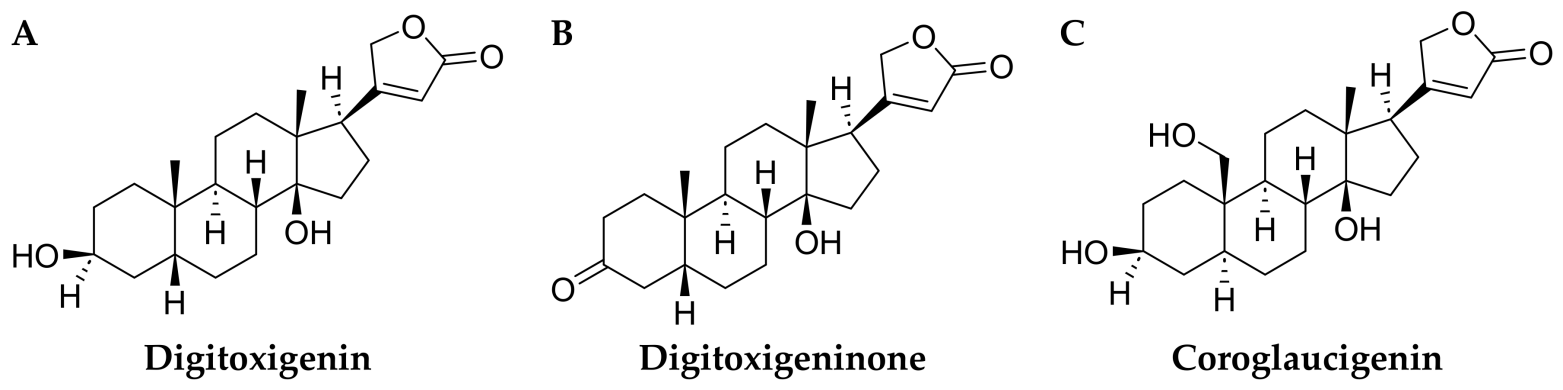
Structures of the used cardenolides digitoxigenin (**A**), digitoxigenone (**B**) and coroglaucigenin (**C**).

Digitoxigenin, digitoxigenone and coroglaucigenin (Figure 9) originate from the estate of Prof. H. T. Andrew Cheung (University of Sydney, Department of Pharmacy) and are managed by the Molecular Archive at the Karlsruhe Institute of Technology. Digitoxigenin is a 5β-cardenolide and the aglycone of the cardiac glycoside digitoxin and is found in the digitalis plants as well. The semi-synthetic cardenolide digitoxigenone is a 3-keto form of digitoxigenin (Figure 9A,B). The natural 5α-cardenolide coroglaucigenin was isolated from *Asclepias vestita* and was, as all used cardenolides, further characterized by 400 MHz nuclear magnetic resonance spectroscopy [50]. Further chemical properties are accessible in the Chemotion Repository [51]; the compounds are fully characterized and the raw data available therein. Cardenolides typically bind the Na^+^/K^+^-ATPase and are partially used in cardiac conditions due to their cardiotonic effects and gained interest in anti-parasitic, anti-inflammatory and cancer research.

For the following experiments, all cardenolides were used at the concentrations of 1, 5, 10, and 25 µM and were dissolved in DMSO.

### 4.2. Human Neuroblastoma (SK-N-SH) Cell Culture

As described in our previous studies [52,53], human SK-N-SH neuroblastoma cells were obtained from the American Type Culture Collection (HTB-11, Rockville, MD, USA) and cultured at 5% CO_2_, 37 °C, in a humidified culture atmosphere. The 1× minimum essential medium (MEM) contained Earl’s salts, 10% fetal bovine serum (Bio & SELL GmbH, Feucht/Nürnberg, Germany), 1 mM L-glutamine, 1 mM sodium pyruvate, 2 mL of 100× MEM vitamin solution, 40 units/mL penicillin, 40 µg/mL streptomycin and 0.1 µg/mL fungizone^®^ (all Gibco, Thermo Fisher Scientific, Bonn, Germany). The cells were passaged with trypsin when grown to about 90% confluency and reseeded with approximately 2 × 10^5^ cells per cm^2^ to 6-, 12-, 24-, or 96-well plates or new cell culture flasks, respectively. In the following day, the medium was changed and cells were used for further experiments.

### 4.3. Cell Viability Assay

Viability of SK-N-SH cells was measured by quantitative colorimetric MTT assay as previously described [52,53]. This assay determines the number of metabolically active cells and allows conclusions about viable cells in the culture based on the reduction of a yellow tetrazolium salt (3-(4,5-dimethylthiazol-2-yl)-2,5-diphenyltetrazolium bromide or MTT; Sigma-Aldrich GmbH, Taufkirchen, Germany) to purple formazan in the cells. Cells (approx. 25 × 10^3^ cells/well) were cultured in 96-well plates for 24 h. Afterwards, the medium was changed, and cells were treated with various concentrations of digitoxigenin, digitoxigenone and coroglaucigenin (1, 5, 10 and 25 µM), with or without 10 U/mL IL-1β for 20 h. 20 µL Ethanol was used as positive control to induce cell death. After this treatment, cells were incubated with 20 µL MTT solution (working concentration 5 mg MTT/mL) for additional 4 h at 37 °C. Next, the medium was removed and 200 µL of DMSO were added to all wells. Thereafter, the colorimetric reaction was measured by the MRX^e^ micro-plate reader (Dynex Technologies, Denkerdorf, Germany) at 595 nm.

### 4.4. Determination of PGE_2_ Release

SK-N-SH cells were pretreated with digitoxigenin, digitoxigenone and coroglaucigenin (1, 5, 10 and 25 µM) for 30 min. In the following step, cells were incubated for another 24 h with or without IL-1β (10 U/mL) and supernatants were collected afterwards. The commercially available enzyme immunoassay (EIA) kit (Cayman Chemicals, Ann Arbor, MI, USA, distributed by BioMol, Hamburg, Germany) was used for measuring levels of PGE_2_ based on the manufacturer’s protocol as described in previous studies [52,53,54,55]. The photometric extinction was measured at 405 nm using the MRX^e^ micro-plate reader. The results were normalized to IL-1β and expressed as the percentage change in PGE_2_ levels from three independent experiments.

### 4.5. Determination of Cytokine Production

SK-N-SH cells were pre-incubated with different concentrations (1, 5, 10, and 25 µM) of digitoxigenin, digitoxigenone and coroglaucigenin for 30 min. Within the next 24 h, cells were treated with or without IL-1β (10 U/mL) and afterwards supernatants were collected. Supernatants were centrifuged at 1000× *g* for 5 min at 4 °C, then the concentrations of IL-6 and IL-8 were measured with commercially available immunoassays (ELISA) kits (cat. Nos. D60508, D8000C Bio-Techne GmbH, Wiesbaden, Germany) based on the manufacturer’s instructions as previously established [53,54]. Briefly, the 96-well microplates were coated with the capture antibody overnight, blocked at the second day, washed and incubated with respective standards and diluted samples. Appropriate dilutions of the supernatants were determined in preliminary tests. In the following steps, detection antibody, Streptavidin-HRP, substrate solution and stop solution were added to every well strictly following the instructions. At the end, photometric extinction was measured at 450 nm by the MRX^e^ micro-plate reader. Based on the standard curves and the measured ODs concentrations were determined. The IL-1β positive control was set as 100% and the other concentrations were normalized to the positive control as percentages.

### 4.6. RNA Isolation and Quantitative PCR

Effects of digitoxigenin, digitoxigenone and coroglaucigenin on the expression of COX-2, mPGES-1, IL-6 and IL-8 in SK-N-SH cells were measured using quantitative PCR (qPCR) as previously described [52,53,54]. First, SK-N-SH cells were incubated with various concentrations of digitoxigenin, digitoxigenone and coroglaucigenin for 30 min and then stimulated with IL-1β (10 U/mL) for 4 h. RNA isolation was performed using the GeneMATRIX Universal RNA Purification Kit (cat. no.: E3598, Roboklon GmbH, Berlin, Germany) based on the manufacturer’s protocol.

Then, cDNA was reverse transcribed from 500 ng of total RNA in a 30 μL total reaction volume with an initial denaturation at 70 °C (10 min) with the following amplification cycle after the addition of the master mix. The qPCR amplification was carried out by the CFX96 real-time PCR detection system (Bio-Rad Laboratories GmbH, Feldkirchen, Germany). Glyceraldehyde 3-phosphate dehydrogenase (GAPDH) served as an internal control for sample normalization. The primer sequences were GAPDH: Forward (Fwd): 5′-TGGGAAGCTGGTCATCAAC-3′/Reverse (Rev): 5′-GCATCACCCCATTTGATGTT-3′, COX-2: Fwd 5′-CTTCACGCATTTCAAG-3′/Rev 5′-TCACCGTAAAGTCCAC-3′, mPGES-1: Fwd 5′-TGCAGCACGCTGCTGGTCAT-3′/Rev 5′-GTCGTTGCGGTGGGCTCTGAG-3′, IL-6: Fwd 5′-CAATGAGGAGACTTGCCTGG-3′/Rev 5′-GCACAGCTCTGGCTTGTTCC-3′, IL-8: Fwd 5′-GTTTTTGAAGAGGGCTGAGAATTC-3′/Rev 5′-ATGAAGTGTTGAAGTAGATTTGCTTG-3′. Primers were designed using Universal ProbeLibrary Assay Design Center (Roche Diagnostics, Mannheim, Germany), Primer-BLAST (Online-Tool; NLM, NCBI, Bethesda, MD, USA) and obtained from biomers.net GmbH (Ulm, Germany).

### 4.7. Determination of COX Enzymatic Activity

The AA assay was performed to assess direct inhibitory effects of digitoxigenin, digitoxigenone and coroglaucigenin on COX enzymatic activity as previously described [52,53,54,55]. Briefly, SK-N-SH cells were seeded to 24-well plates, the medium was removed and replaced with a serum-free medium after 24 h. The three cardenolides digitoxigenin, digitoxigenone and coroglaucigenin (1, 5, 10, and 25 µM) and the selective inhibitor of COX-1 SC560 [(1 and 10 µM); Sigma-Aldrich GmbH, Taufkirchen, Germany] were added to the respective wells and incubated for 15 min. Afterwards, 30 µM AA (Sigma-Aldrich GmbH, Taufkirchen, Germany) was added and incubated for another 15 min. Supernatants were collected and used for the determination of PGE_2_ as described before.

COX-2 activity was measured as described for COX-1, but COX-2 was previously induced by adding IL-1β (10 U/mL) for 24 h to all cells. Diclofenac sodium [(0.1 and 1 µM); Sigma-Aldrich GmbH, Taufkirchen, Germany] was used as commercial COX-2 preferential inhibitor.

### 4.8. Western Blot

Western blots were conducted as previously published [52,53,54,55]. SK-N-SH were stimulated with IL-1β (10 U/mL) for 30 min (signaling) or 24 h (COX-2, mPGES-1) after pretreatment with digitoxigenin, digitoxigenone and coroglaucigenin (1, 5, 10 and 25 µM) for 30 min. After stimulation, cells were washed with cold phosphate-buffered saline (PBS) and lysed with lysis buffer (42 mM Tris-HCl, 1.3% sodium dodecyl sulfate (SDS), 6.5% glycerin, 100 µM sodium orthovanadate, and 2% phosphatase and protease inhibitors). Protein concentrations of the samples were measured using the bicinchoninic acid protein (BCA) assay kit (Thermo Fisher Scientific, Bonn, Germany) according to the manufacturer’s instructions. For Western blots, 20 µg of total protein from each sample was subjected to SDS-PAGE under reducing conditions. Afterward, proteins were transferred onto polyvinylidene fluoride (PVDF) membranes (Merck Millipore, Darmstadt, Germany). After blocking with 5% milk solution (Bio-Rad Laboratories GmbH, Feldkirchen, Germany) in Tris-buffered saline (TBS) containing 0.1% Tween 20 (TBS-T), membranes were incubated with primary antibodies. Primary antibodies used were goat anti-COX-2 (sc-1745, Santa Cruz Biotechnology Inc., Heidelberg, Germany; 1:500), rabbit anti-mPGES-1 (No. 160140, Cayman Chemical Co., Ann Arbor, MI, USA; 1:6000), anti-p42/44 MAPK (Erk 1/2) (9102S, Cell Signaling Technology Inc., Danvers, MA, USA; 1:1000), anti-p38 MAPK (9212S, Cell Signaling Technology Inc., Danvers, MA, USA; 1:1000), anti-SAPK/Jnk (9252S, Cell Signaling Technology Inc., Danvers, MA, USA; 1:1000), anti-phospho p38 MAPK (9211S, Cell Signaling Technology Inc., Danvers, MA, USA; 1:1000), anti-phospho SAPK/Jnk (9251S, Cell Signaling Technology Inc., Danvers, MA, USA; 1:1000), anti-phospho p42/44 MAPK (Erk 1/2) (9101S, Cell Signaling Technology Inc., Danvers, MA, USA; 1:1000), phospho-NF-κB p65 (C-20) (sc-372, Santa Cruz Biotechnology, Inc., Heidelberg, Germany; 1:1000), Vinculin (AB6039, Merck, Darmstadt, Germany, 1:20,000), anti-phospho IκB (sc-8404, Santa Cruz Biotechnology Inc., Heidelberg, Germany; 1:500), and mouse anti-Vinculin (cat. no.: V9264, 1:20,000, Sigma-Aldrich GmbH, Taufkirchen, Germany). Primary antibodies were diluted in TBS-T and 5% BSA. Membranes were incubated with the primary antibody overnight at 4 °C, followed by incubation with the respective secondary antibodies. After extensive washing (three times for 15 min each in TBS containing 0.1% Tween 20), proteins were detected with horseradish peroxidase (HRP)-coupled anti-goat IgG (Santa Cruz Biotechnology Inc., Heidelberg, Germany, 1:10,000) and anti-rabbit IgG (RD systems, Wiesbaden, Germany, 1:10,000) using enhanced chemiluminescence (ECL) reagents (Biozym, Hessisch Oldendorf, Germany) and captured using the ChemiDoc MP imaging system (Bio-Rad Laboratories GmbH, Feldkirchen, Germany). Equal protein loading and transfer were assessed by subjection of each sample to a Western blot for vinculin or the corresponding total protein for signaling. All Western blot experiments were carried out three times. Densitometric analysis was performed using ImageJ software (V1:48t, NIH, Bethesda, MD, USA).

### 4.9. Statistical Analysis

Raw values were converted to percentage considering IL-1β (10 U/mL) or the appropriate positive control, such as untreated cells for the MTT assay, as 100%. Values of all experiments are presented as mean ± SD of at least three independent experiments. Values were compared using one-way ANOVA with Dunett’s post hoc test (Prism 10 software, GraphPad Software Inc., San Diego, CA, USA). The level of significance was set at * *p* < 0.05, ** *p* < 0.01, *** *p* < 0.001, and **** *p* < 0.0001.

## 5. Conclusions

In conclusion, this study provides novel evidence supporting the anti-neuroinflammatory potential of cardenolides in IL-1β-activated neuronal cells. Our findings demonstrate that these compounds effectively suppress the AA/COX-2/PGE_2_ pathway and pro-inflammatory cytokine production with digitoxigenin showing the strongest effects of the tested cardenolides. While the inhibition of p38 MAPK and NF-κB signaling by digitoxigenin suggests a potential mechanism for this specific derivative, the potent anti-inflammatory effects of digitoxigenone and coroglaucigenin, which are independent of these pathways, highlight the existence of additional, distinct molecular targets within the cardenolide family. Collectively, by targeting key inflammatory enzymes and mediators through both shared and compound-specific mechanisms, cardenolides represent promising therapeutic candidates for mitigating neuroinflammation-associated neurodegenerative processes. Future in vivo studies and clinical evaluations are warranted to further explore their efficacy, safety, and precise mechanisms of action in the context of central nervous system disorders.

## Data Availability

The studied cardenolides originate from the estate of H. T. Andrew Cheung (University of Sydney, Department of Pharmacy), which is managed by the Molecular Archive at the Karlsruhe Institute of Technology. Chemical analyses are available via the Chemotion Repository (https://www.chemotion-repository.net/, accessed on 29 July 2026) and can be accessed via the following collection DOI: https://dx.doi.org/10.14272/collection/CWG_2026-07-29 [46]. The biological data presented in this manuscript are available from the corresponding author upon request.

## Supplementary Materials

The following supporting information can be downloaded at: https://www.mdpi.com/article/10.3390/molecules31193501/s1, Figure S1: Effects of digitoxigenone and coroglaucigenin on NF-κB and p38 MAPK phosphorylation. Effects of digitoxigenone (A,B) and coroglaucigenin (C,D) on phosphorylation of NF-κB (A,C) and p38 MAPK (B,D) in IL-1β-stimulated SK-N-SH cells. Cells were stimulated as described before. Values are presented as the mean ± SD of three independent experiments. Statistical analysis was performed using one-way ANOVA with Dunnett’s post hoc tests compared to IL-1β.
