# Supplementary material for: Anti-Neuroinflammatory Effects of Cardenolides in IL-1β-Activated SK-N-SH Cells
Source: Molecules. 2026 Oct 1;31(19):3501. doi: 10.3390/molecules31193501 (PMC13666663; doi:10.3390/molecules31193501)

Figure S1. Effects of digitoxigenone and coroglaucigenin on NF-κB and p38 MAPK phosphorylation. Effects of digitoxigenone (A,B) and coroglaucigenin (C,D) on phosphorylation of NF-κB (A,C) and p38 MAPK (B,D) in IL-1β-stimulated SK-N-SH cells. Cells were stimulated as described before. Values are presented as the mean ± SD of three independent experiments. Statistical analysis was performed using one-way ANOVA with Dunnett's post hoc tests compared to IL-1β.

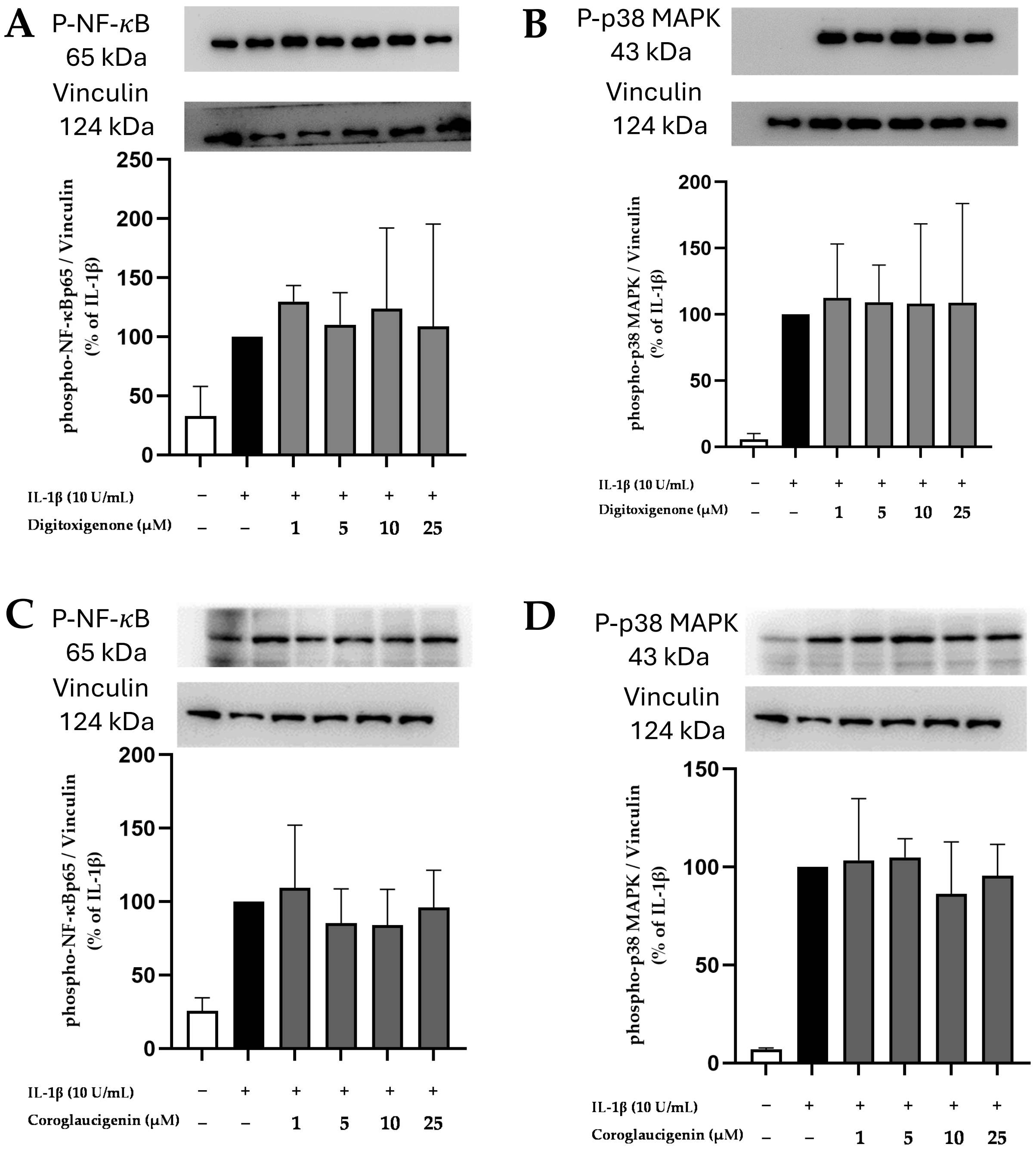

Supplement: Supplementary file 1 [file molecules-31-03501-s001.zip › molecules-4566778-supplementary.pdf]
